# Pepsinogen I and II expressions in situ and their correlations with serum pesignogen levels in gastric cancer and its precancerous disease

**DOI:** 10.1186/1472-6890-13-22

**Published:** 2013-09-02

**Authors:** Ping Li, Caiyun He, Liping Sun, Nannan Dong, Yuan Yuan

**Affiliations:** 1 Tumor Etiology and Screening Department of Cancer Institute and General Surgery, Liaoning Provincial Education Department, The First Affiliated Hospital of China Medical University, Key Laboratory of Cancer Etiology and Prevention(China Medical University), 155# North Nanjing Street, Heping District, Shenyang City 110001, Liaoning Province, China

**Keywords:** Pepsinogen, Gastric disease, Correlation

## Abstract

**Background:**

Serum pepsinogen (PG) I/II ratio has been widely used as “serological biopsy” for the screening of gastric cancer (GC) and atrophic gastritis (GA). However, study concerning in situ expression of PGs is currently insufficient, particularly for their relationship with serum PGs levels. This study was designed to investigate in situ expression of PGI and PGII in subjects with normal mucosa (NOR), superficial gastritis (GS), GA and GC, and to evaluate the correlations between PGs expressions in situ and in serum.

**Methods:**

185 subjects were enrolled for the study, including 30 NOR, 70 GS, 54 GA and 31 GC. PGI and PGII expressions in situ and in serum were detected by immunohistochemistry and enzyme-linked immunosorbent assay (ELISA) respectively. *H. pylori* immunoglobulin (Ig) G was also determined by ELISA.

**Results:**

In situ expressions of PGI, PGII and PGI/II ratio consistently decreased in sequence of NOR/GS- > GA- > GC. The expressions of PGI, PGII and PGI/II ratio in situ were statistically higher in youngers than in olders (P < 0.05). In the NOR subjects, PGI staining was statistically higher in males than that in females (p = 0.02). For the correlations between in situ and serum expressions of PGI, PGII and PGI/II ratio, a borderline correlation in the total study sample (r = 0.131, P = 0.076) and a statistical correlation in GA cases (r = 0.307, P = 0.027) were observed for the PGI/II ratio. The PGI expression correlated well with that of PGII in situ and in serum.

**Conclusions:**

The in situ levels of PGI, PGII and PGI/II ratio sharply decreased in the GA and GC cases. The youngers exhibited higher levels of PGI, PGII and PGI/II ratios than the olders. The in situ PGI/II ratio rather than PGI and PGII alone showed certain correlation with that in serum, and the PGI expression correlated well with PGII expression. Further studies with large-scale samples are still required to validate our findings.

## Background

Human gastric mucosa contains two abundant and distinguishable aspartic proteinases, namely pepsinogen I (PGI or PGA) and pepsinogen II (PGII or PGC) [1]. The majority of PGs are present in gastric mucosa and a small part of those may be released into blood [2]. Typically, PGs are present as zymogens in gastric mucosa and can be converted into active proteolytic forms under certain acidic condition in stomach lumen. The activated pepsins are extremely important for the digestive process in stomach [1].

There are overwhelming epidemiological evidences supporting that serum level of PGI and/or PGI/II ratio correlates well with morphologic and functional changes of gastric mucosa [3-7]. Accordingly, they have been widely used as ‘serological biopsy’ for the screening of gastric cancer (GC) and its precancerous lesions [3-7]. In spite of the wide use of serum PGs in clinical practice, study concerning in situ expressions of PGs, particularly in the stepwise progression from normal mucosa (NOR), superficial gastritis (GS), atrophic gastritis (GA) to carcinoma, is currently insufficient. The questions whether the PGs expression changes in situ are synchronistic with those in serum and which factors affect the in situ expression of PGs are still not resolved based on previous studies. These may, to some extent, puzzle the clinical work on how to appropriately interpret the variations of serum PGs expression in different status of gastric diseases.

The present study was conducted to investigate in situ expressions of PGI, PGII and PGI/II ratio in the sequence of NOR- > GS- > GA- > GC. The possible influences of sex, age and *H. pylori* infection on PGs expressions in situ were also explored. Moreover, the correlations between in situ and serum expression of PGs and between PGI and PGII expression in situ and in serum were evaluated.

## Methods

### Patients

A total of 185 subjects (male 110 and female 75) were enrolled in this study, including NOR (n = 30), GS (n = 70), GA (n = 54), and GC (n = 31). All the subjects were retrospectively enrolled from a health check program for gastric cancer screening in Zhuanghe county of Liaoning province, China between 1998 and 2010. The diagnosis of gastric disease was established by gastroscopic examination and confirmed by histopathology. Histopathological findings were assessed according to the Consensus on Chronic Gastritis formulated at the National Symposium in combination with the updated Sydney System and the World Health Organization (WHO) criteria [8-10]. The NOR individuals were confirmed to have relative normal gastric mucosa without evidence of *H. pylori* infection or gastrointestinal symptom. The GS subjects have only slight or moderate superficial gastritis without atrophic or intestinal metaplasia lesions. The GA cases have atrophic gastritis with or without intestinal metaplasia. Information of sex, age was retrospectively extracted from registered documents.

This cross-sectional study was approved by the Human Ethics Review Committee of China Medical University. Written informed consents were obtained from the participants.

### Immunohistochemistry staining of PGI and PGII

For the retrieval of antigens, detection was performed in 5 μm-thick sections from sequentially sliced samples of paraffin-embedded specimens. Dewaxing sections were heated in citrate buffer (pH 6.0) using a microwave for 10 min. Overnight incubation at 4°C was carried out for the binding of primary antibodies (PGI, anti-pepsinogen A antibody, trade name: 2 F5, 1:600 dilution; PGII, anti-pepsinogen C antibody, trade name: 2D5, 1:400 dilution; both antibodies were donated by Japan Clinical Inspection Institute) [11,12]. Afterwards, SP-two step immunostaining was performed according to the instructions of the kit (Kit-9801D2 from Maixin Company in Fujian, China). For all stainings, positive controls were carried out, and staining was accepted only if controls showed evaluable results.

### Assessment of immunohistochemical staining

Immunohistochemical results were judged by IRS (immunoreactive score), which was determined by two independent observers. The IRS was calculated using two indexes of staining intensity (SI) and the percentage of positive cells (PP). The SI in cytoplasm was graded as: 0 = no, 1 = weak, 2 = moderate, 3 = strong staining. The PP was categorized as: 0 = no stained cells, 1 = stained cells < 25%, 2 = stained cells 25 ~ 50%, 3 = stained cells 51% ~ 75%; 4 = stained cells > 75%. For each sample an IRS was calculated as SI × PP with a possible maximum score of 12. The assessment result was defined as either negative (0), weakly positive (1 ~ 3), positive (4 ~ 7) or strongly positive (8 ~ 12).

In the present study, PGI staining was located in gastric corpus glands while PGII staining was located in both gastric corpus and antral mucosa. Therefore, PGI IRS was only assessed in the gastric corpus mucosa, and PGII IRS was assessed in both corpus and antral mucosa for each participant. For PGI staining, we evaluated the stained status of all the chief cells of gastric corpus glands for NOR and GS subjects, the remaining chief cells of subsistent corpus glands for GA subjects, and cells of the cancerous lesions for GC subjects. For PGII staining, we evaluated the staining status of all the gastric corpus and antrum glands for NOR and GS subjects, the remaining gastric corpus and antral glands for GA subjects, and cells of the cancerous lesion for GC subjects.

### Test for *H. Pylori* serology

The detailed method of examination of *H. pylori* serology has been described in our previous study [13]. In brief, about five ml of fasting venous blood was collected from each participant. The serum sample was obtained after centrifugation at 3000 × g for 10 minutes. Serum Immunoglobin (Ig) G antibodies of *H. pylori* were detected by enzyme-linked immunosorbent assay (ELISA) kit (Biohit Co., Ltd., Helsinki, Finland) according to the manufacturer's instructions. A reading of *H. pylori*-IgG higher than 34 EIU (enzyme immune-units) was regarded as *H. pylori* seropositive.

### Test for serum PGI and PGII expression

The detailed method of examination of serum pepsinogens has been described in our previous study [14]. Approximately 5 ml fasting blood was collected from each participant. The blood was centrifuged at 3000 × g for 10 minutes and the serum was stored immediately at −20°C until used. Serum PGI (sPGI) and PGII (sPGII) concentrations (microg/L) were detected by ELISA kit (Biohit Co., Ltd., Finland) according to the manufacturer's instructions. Five percent of all samples were assayed in duplicate.

### Statistical analysis

All statistical analysis was performed using the SPSS (13.0) program (SPSS, Chicago, USA). The distributions of discrete variables were represented as frequencies and percentages. The averages of continuous variables were represented as median (25%, 75%). The positive rates of PGI and PGII staining in different gastric diseases were compared by Pearson's Chi-square test or Fisher’s exact test. The correlation coefficient between two variables was measured by partial correlation controlling for sex and age. The IRS of in situ expression of PGI, PGII and PGI/II ratio among multiple groups were compared by the Kruskal–Wallis test; if statistical significance (P < 0.05) was indicated, the difference between two groups was further tested by the Mann–Whitney *U*-test. A two-tailed *P* value less than 0.05 was considered as statistically significant.

## Result

### Dynamic in situ expression of PGs in different gastric tissues

**Figure 1:**
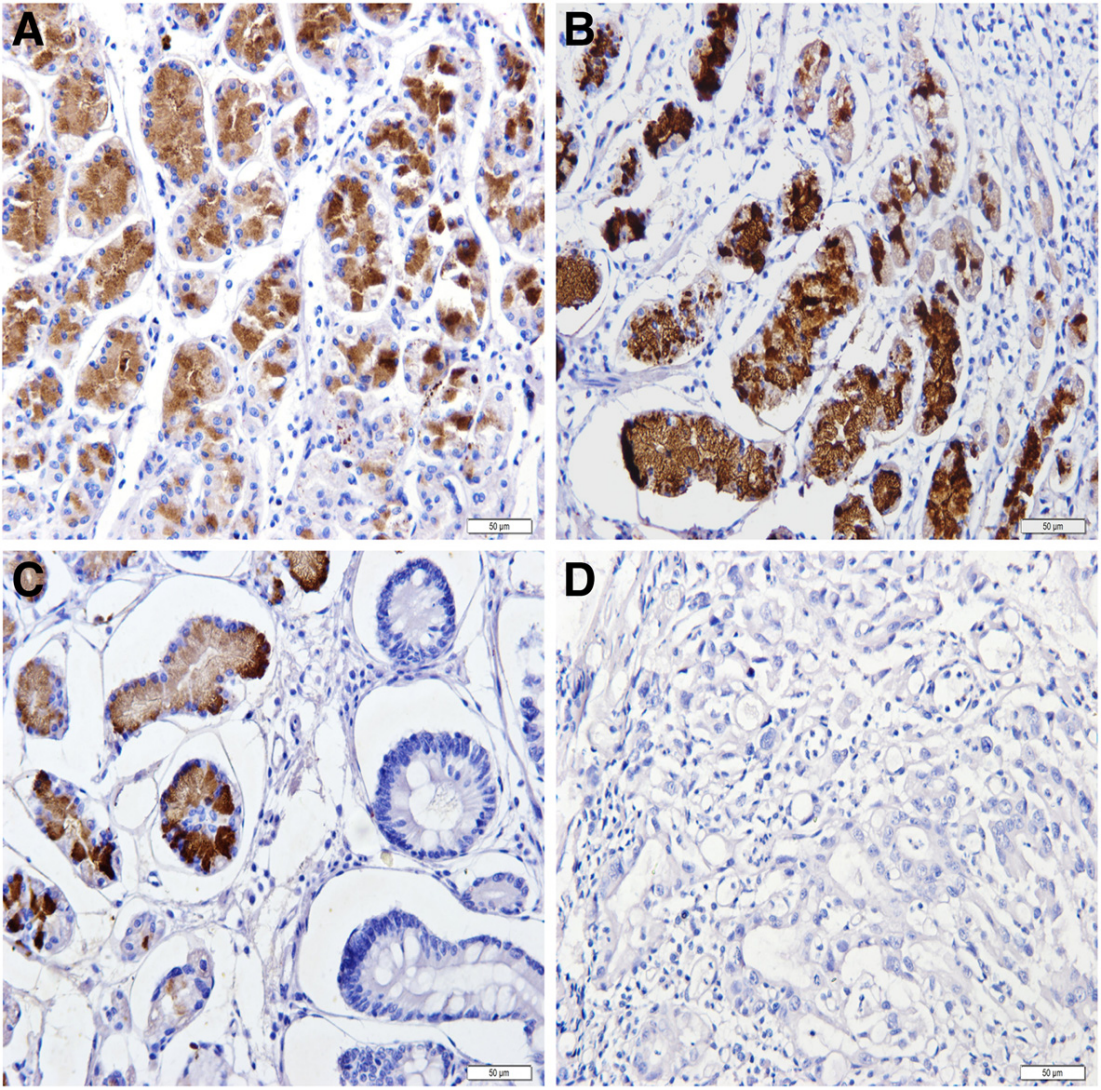
**Expression of PGI in corpus glands in different gastric tissues (immunohistochemical staining × 200). (A)** NOR mucosa; **(B)** GS mucosa; **(C)** GA mucosa; **(D)** GC mucosa.

**Figure 2:**
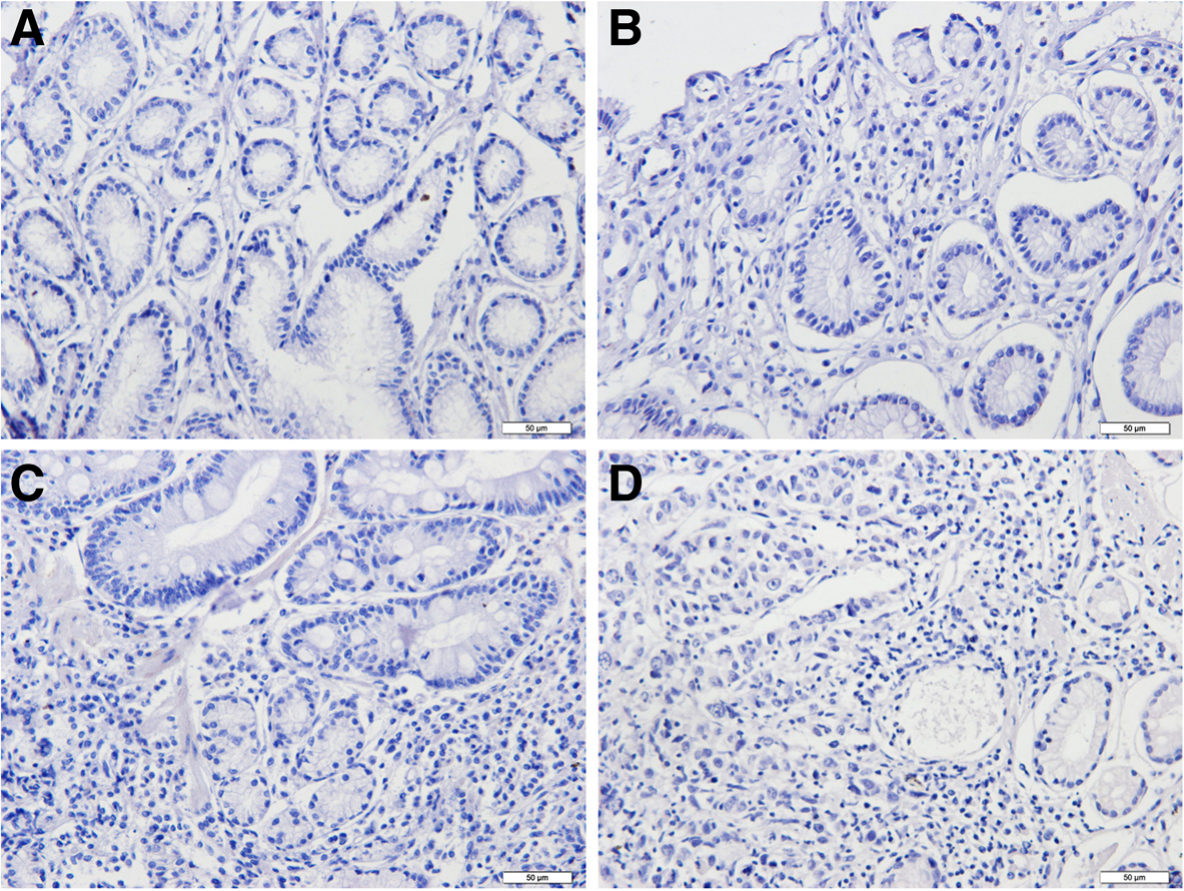
**Negative expression of PGI in all antral glands in different gastric tissues (immunohistochemical staining × 200). (A)** NOR mucosa; **(B)** GS mucosa; **(C)** GA mucosa; **(D)** GC mucosa.

**Figure 3:**
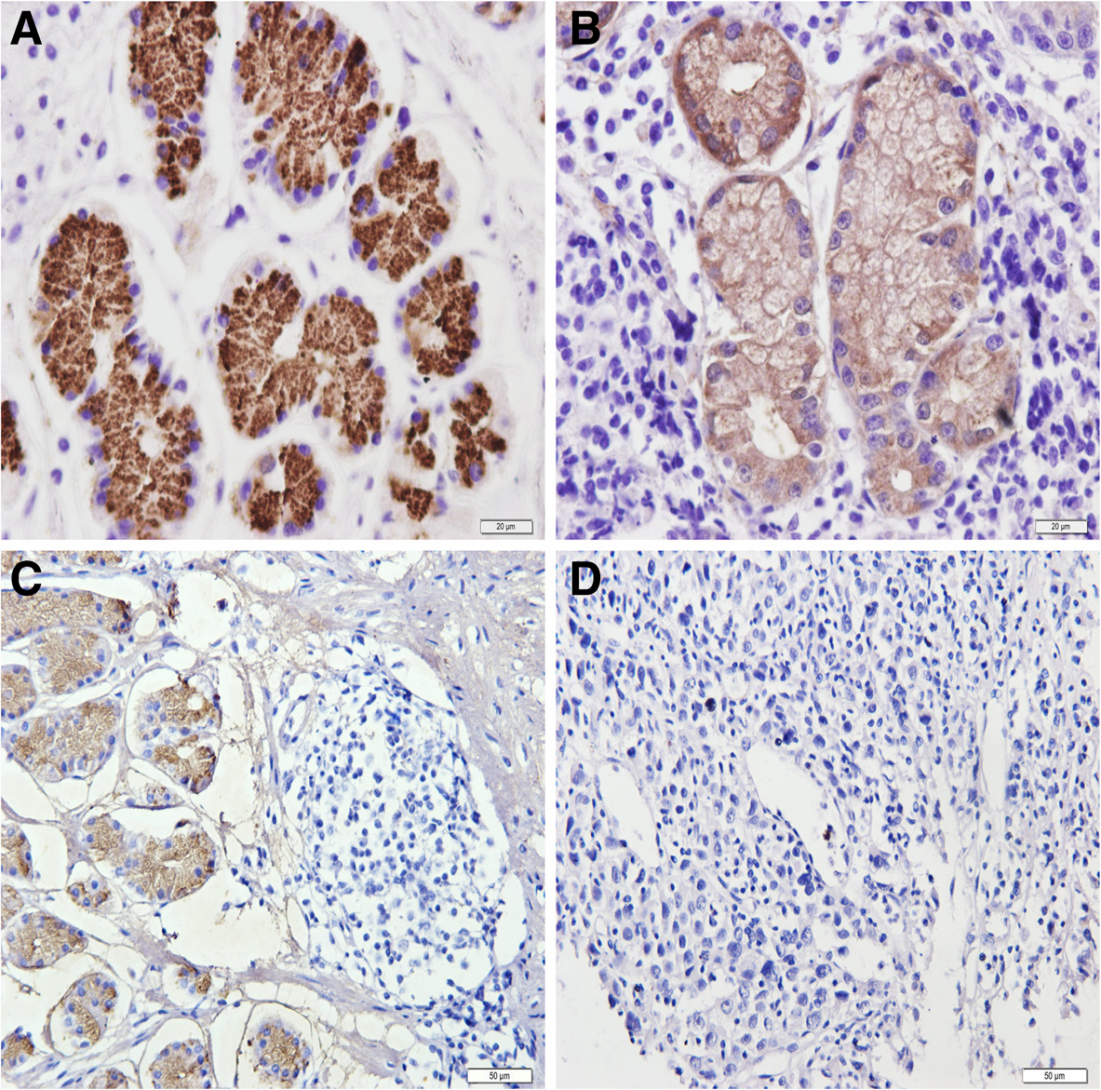
**Expression of PGII in corpus glands in different gastric tissues (immunohistochemical staining × 200). (A)** NOR mucosa; **(B)** GS mucosa; **(C)** GA mucosa; **(D)** GC mucosa.

**Figure 4:**
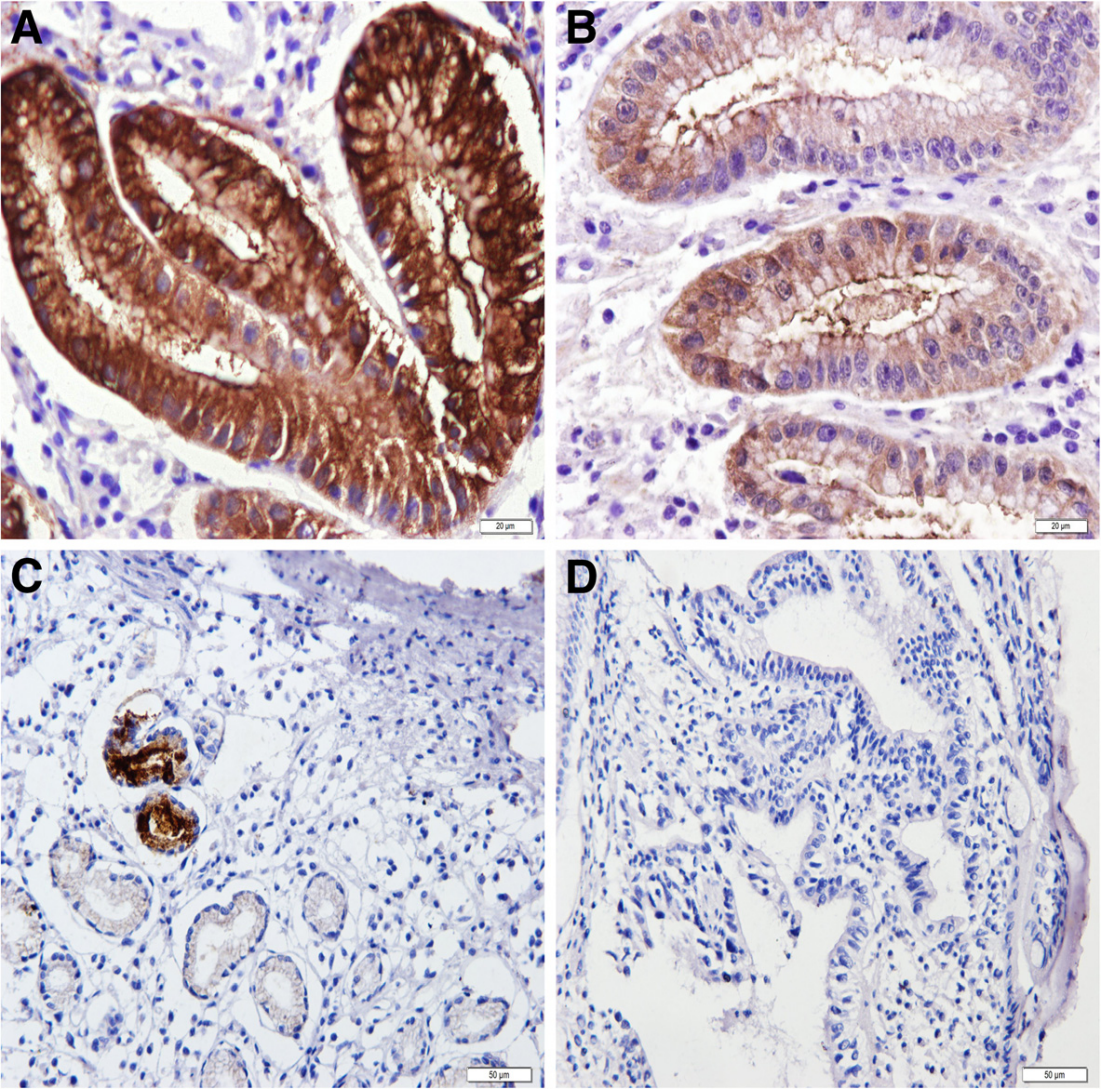
**Expression of PGII in antral glands in different gastric tissues. (A)** NOR mucosa (immunohistochemical staining × 400); **(B)** GS mucosa (immunohistochemical staining × 400); **(C)** GA mucosa (immunohistochemical staining × 200); **(D)** GC mucosa (immunohistochemical staining × 200).

Both PGI and PGII staining were located in the cytoplasm and cell membrane of gastric epithelial cells but in different part of stomach. Staining for PGI was positive in corpus mucosa but negative in all antral mucosa regardless of the status of gastric mucosa (Figures 1 and 2). PGII staining was present in both corpus and antral mucosa (Figures 3 and 4). None of the intestinal metaplasia cells exhibited the PGI or PGII staining. As demonstrated in Table 1, along the sequence of NOR/GS- > GA- > GC, the positive rates and strongly-positive rates of both PGI and PGII expression showed significantly decreased tendencies.

**Table 1:** In situ expression of PGs in different gastric mucosa

	**N**	**Positive rate**	**P value**			**Strongly-positive rate**	**P value**			**in situ PGI/II ratio**^**a**^	**P value**		
			**vs. NOR**	**vs. GS**	**vs. GA**		**vs. NOR**	**vs. GS**	**vs. GA**		**vs. NOR**	**vs. GS**	**vs. GA**
In situ PGI expression													
NOR	30	100.00%				50.00%				1.0(0.7,1.1)			
GS	70	100.00%	/			32.90%	**0.021**^**b**^			1.0(0.7,1.4)	0.292^d^		
GA	54	83.30%	**0.023**^**c**^	**<0.001**^**c**^		3.70%	**<0.001**^**b**^	**<0.001**^**b**^		0.8(0.4,1.1)	0.086^d^	**0.005**^**d**^	
GC	31	0.00%	**<0.001**^**b**^	**<0.001**^**b**^	**<0.001**^**b**^	0.00%	**<0.001**^**b**^	**<0.001**^**b**^	**<0.001**^**b**^	0.0(0.0,0.0)	**<0.001**^**d**^	**<0.001**^**d**^	**<0.001 **^**d**^
In situ PGII expression													
NOR	30	100.00%				63.30%				/			
GS	70	100.00%	/			31.40%	**0.015**^**b**^			/			
GA	54	92.60%	0.291^c^	**0.034**^**c**^		1.90%	**<0.001**^**b**^	**<0.001**^**b**^		/			
GC	31	0.00%	**<0.001**^**b**^	**<0.001**^**b**^	**<0.001**^**b**^	0.00%	**<0.001**^**b**^	**<0.001**^**b**^	**<0.001**^**b**^	/			

^a^, the ratios of PGI IRS to PGII IRS were represented as median (25%, 75%); ^b^, the positive rates and strongly-positive rates of PGI and PGII staining in different gastric diseases were compared by Pearson's Chi-square test; ^c^, the positive rates of PGI and PGII expression in different gastric diseases were determined by Fisher’s exact test; ^d^, the in situ PGI/II ratios among multiple gastric diseases were first tested by Kruskal–Wallis test. If the result was indicated as statistically significant, the Mann–Whitney test was performed to further test the different between two groups. Analyses results with P < 0.05 were highlighted in bold characters.

To further investigate the relative expression of PGI to PGII in situ, the ratio of PGI IRS to PGII IRS was calculated, namely in situ PGI/II ratio. We found that in situ PGI/II ratios also showed a decreased tendency in the sequence of NOR/GS- > GA- > GC, showed corresponding average level of 1.0/1.0, 0.8 and 0 (All the IRS of PGI and PGII staining in GC tissue were zero). In situ PGI/II ratios in GA were statistically lower than those in GS (P = 0.005).

### In situ expression of PGs in different status of sex, age and *H. pylori* infection

The differences of the IRS of PGs staining between females and males, between youngers (age < 50 years) and olders (age ≥ 50 years), and between *H. pylori* seropositive and seronegative subpopulations were evaluated in the total study sample and in the NOR, GS and GA subgroups (Table 2). Of the total study sample, in situ levels of PGI, PGII and PGI/II ratio were observed to be statistically higher in the youngers than in the olders. There were no statistical differences between the youngers and the olders in the subgroups of NOR, GS or GA. In addition, in situ PGI expression was found to be statistically higher in males than that in females in the NOR subgroup.

**Table 2:** **In situ expression of PGs in different sex, age and *H. pylori *status**

	**Total**		**NOR**		**GS**		**GA**	
	**IRS score**^**a**^		**IRS score**^**a**^		**IRS score**^**a**^		**IRS score**^**a**^	
In situ PGI staining								
Sex		0.265		**0.002**		0.599		0.964
Female	4.0(2.0,7.0)		5.0(4.0,7.5)		6.0(4.0,9.0)		2.0(1.0,3.3)	
Male	3.0(0.0,7.0)		9.0(7.0,10.5)		6.0(2.5,8.5)		2.0(1.0,3.0)	
Age(years)		**0.003**		0.681		0.183		0.563
<50	4.0(2.0,9.0)		7.5(5.5,9.3)		7.0(3.0,10.0)		2.0(1.0,4.0)	
≥50	3.0(0.0,6.0)		7.5(5.0,10.8)		5.0(3.0,7.0)		2.0(1.0,3.0)	
*H. pylori* IgG		0.359		/		0.515		0.659
EIU < 34	3.0(1.0,6.0)		7.5(5.0,10.0)		5.0(3.0,9.3)		2.0(1.0,3.0)	
EIU ≥ 34	2.0(0.0,6.0)		/		6.0(4.0,8.8)		2.0(1.0,3.5)	
In situ PGII staining								
Sex		0.073		0.363		0.245		0.643
Female	3.0(1.0,7.0)		7.0(4.5,11.0)		6.0(4.0,9.0)		3.0(1.0,5.0)	
Male	5.0(2.0,7.0)		9.0(7.5,10.0)		5.0(3.0,9.0)		2.0(1.3,4.0)	
Age(years)		**0.003**		0.198		0.260		0.173
<50	5.0(2.0,9.0)		7.5(4.0,9.0)		6.5(3.3,9.8)		3.0(2.0,5.8)	
≥50	3.0(0.0,6.3)		9.5(6.0,11.0)		5.0(2.0,8.0)		2.0(1.0,4.0)	
*H. pylori* IgG		0.942		/		0.344		0.069
EIU < 34	3.0(1.0,6.0)		8.5(5.8,10.3)		6.0(2.0,9.0)		2.0(1.0,3.0)	
EIU ≥ 34	3.0(0.0,6.0)		/		5.5(4.0,9.8)		3.0(2.0,5.5)	
In situ PGI/PGII staining								
Sex		0.453		0.123		1.000		0.650
Female	0.8(0.4,1.3)		0.8(0.5,1.0)		1.0(0.7,1.5)		0.6(0.2,1.2)	
Male	0.8(0.0,1.1)		1.0(0.8,1.2)		1.0(0.7,1.5)		0.8(0.4,1.0)	
Age(years)		**0.011**		0.530		0.864		0.462
<50	1.0(0.6,1.3)		1.0(0.6,1.6)		1.0(0.7,1.4)		1.0(0.4,1.0)	
≥50	0.7(0.0,1.0)		01.0(0.8,1.0)		1.0(0.7,1.5)		0.7(0.1,1.0)	
*H. pylori* IgG		0.062		/		0.763		0.599
EIU < 34	0.9(0.3,1.4)		1.0(0.7,1.1)		1.0(0.7,1.5)		0.8(0.2,2.5)	
EIU ≥ 34	0.6(0.0,1.0)		/		1.0(0.7,1.4)		0.7(0.4,1.0)	

^a^, IRS of PGI, PGII and PGI/II ratio were represented as median (25%, 75%); ^b^, in situ expression of PGI, PGII, PGI/II ratio between two groups were compared by Mann–Whitney test. Analyses results with P < 0.05 were highlighted in bold characters.

### Correlations between PGs expression in situ and in serum

**Figure 5:**
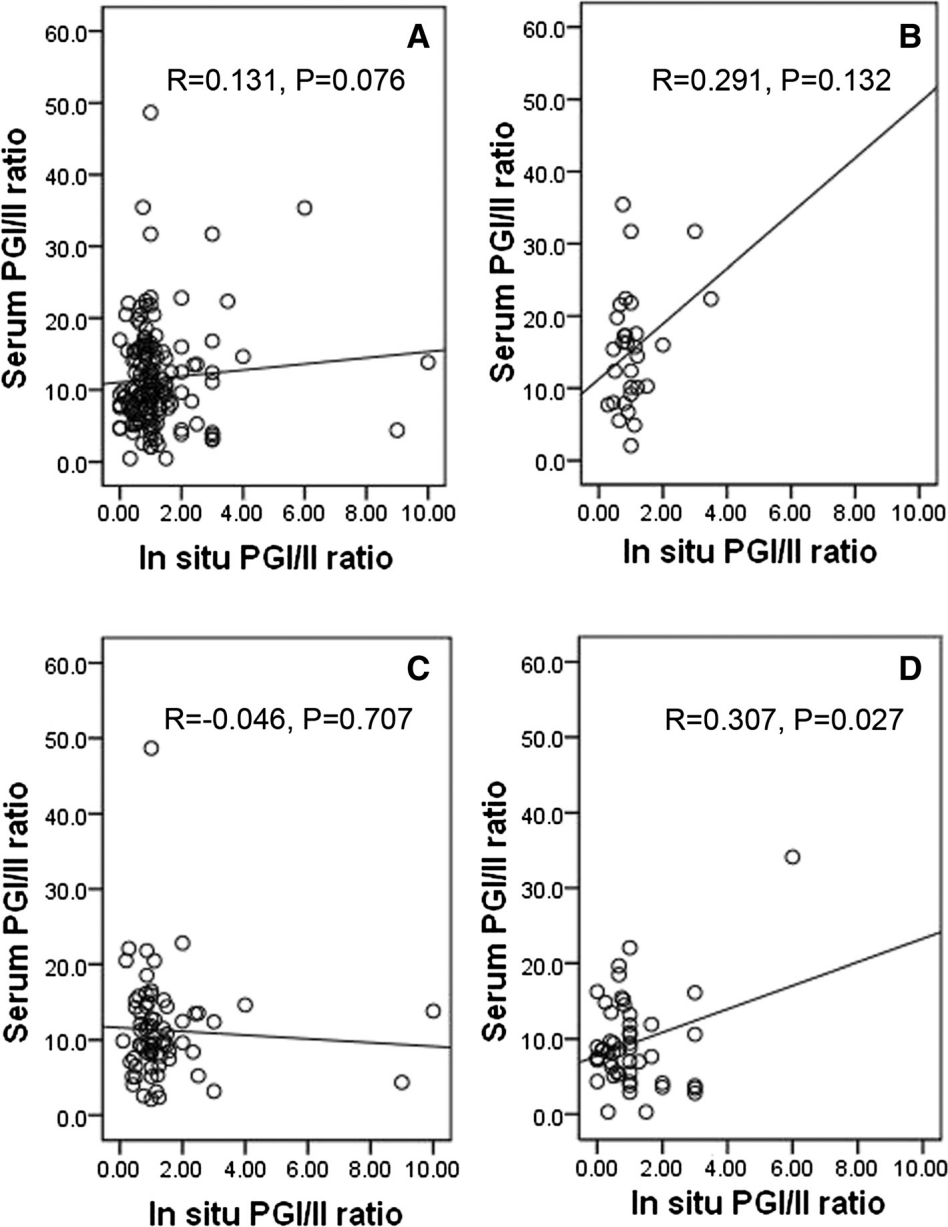
**Scatter plots of correlations between in situ PGI/II ratio and serum PG/II ratio. (A)** correlation between in situ PGI/II ratio and serum PGI/II ratio in total study sample; **(B)** correlation between in situ PGI/II ratio and serum PGI/II ratio in NOR subgroup; **(C)** correlation between in situ PGI/II ratio and serum PGI/II ratio in GS subgroup; **(D)** correlation between in situ PGI/II ratio and serum PGI/II ratio in GA subgroup.

**Figure 6:**
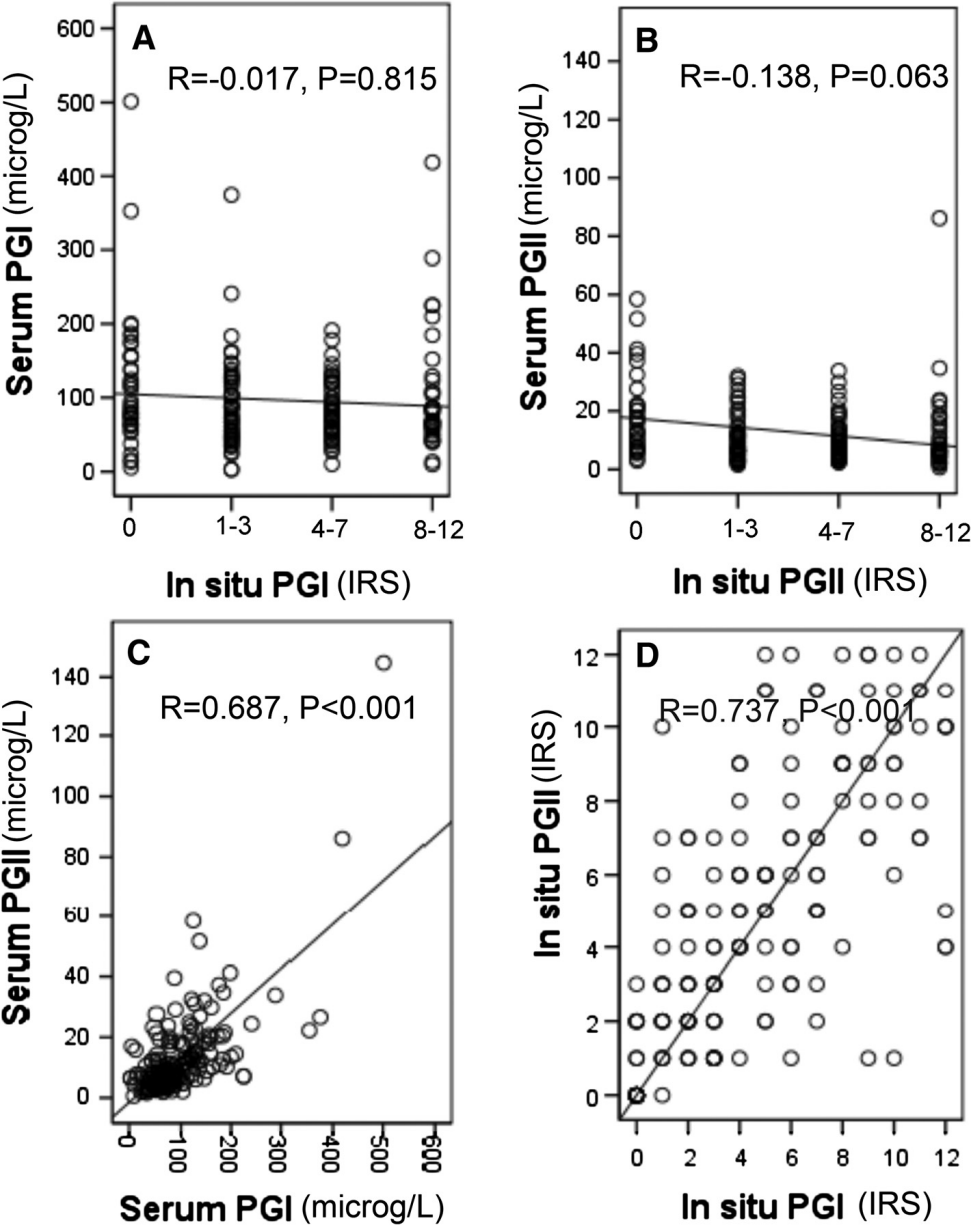
**Scatter plots of correlations between pepsinogens. (A)** correlation between in situ PGI (IRS scores were divided into four subgroup, i.e. 0, 1–3, 4–7, 8–12) and serum PGI in total study sample; **(B)** correlation between in situ PGII (IRS scores were divided into four subgroup, i.e. 0, 1–3, 4–7, 8–12) and serum PGII in total study sample; **(C)** correlation between serum PGI and serum PGII in total study sample; **(D)** correlation between in situ PGI (IRS score) and in situ PGII (IRS score) in total study sample.

The expression levels of PGI, PGII and PGI/II ratio in situ and in serum were summarized in Table 3. We explored the correlations between in situ and serum levels of PGI, PGII and PGI/II ratio (Table 4). A borderline correlation in the total study sample (r = 0.131, P = 0.076) and a statistical correlation in GA cases (r = 0.307, P = 0.027) were observed for the correlation between in situ and serum levels of PGI/II ratio (Figure 5). However, we found no statistical correlation between in situ and serum expression of PGI or PGII in the total study sample or in the subgroups of different diseases (all P > 0.05) (Figure 6).

**Table 3:** Expression of PGI, PGII and PGI/II ratio in situ and in serum

**Gastric mucosa**	**N**	**In situ PGI (IRS)**	**In situ PGII (IRS)**	**In situ PGI/II ratio**	**Serum PGI (microg/L)**	**Serum PGI (microg/L)**	**Serum PGI/II ratio**
NOR	30	7.5(5.0,10.0)	8.5(5.8,10.3)	1.0(0.7,1.1)	68.2(47.8,146.8)	6.3(4.1,9.2)	15.3(8.9,20.2)
GS	70	6.0(3.0,9.00	6.0(3.0,9.0)	1.0(0.7,1.4)	72.5(53.5,102.3)	6.4(4.3,13.9)	9.8(7.6,14.3)
GA	54	2.0(1.0,3.0)	2.5(1.0,4.3)	0.8(0.4,1.1)	87.4(56.3,127.1)	10.7(6.6,15.6)	8.6(4.8,12.4)
GC	31	0.0(0.0,0.0)	0.0(0.0,0.0)	0.0(0.0,0.0)	86.6(64.8,139.2)	15.8(7.7,24.9)	6.1(3.7,11.7)

Note: levels of PGI, PGII and PGI/II ratio were represented as median (25%,75%).

**Table 4:** Correlation between PGs expression in situ and in serum

	**In situ PGI and serum PGI**		**In situ PGII and serum PGII**		**In situ PGI/II and serum PGI/II**		**In situ PGI and In situ PGII**		**Serum PGI and Serum PGII**	
	**R**	**P value**	**R**	**P value**	**R**	**P value**	**R**	**P value**	**R**	**P value**
Total	−0.017	0.815	−0.138	0.063	0.131	0.076	0.737	**<0.001**	0.687	**<0.001**
NOR	0.135	0.492	0.124	0.529	0.291	0.132	0.340	0.076	0.766	**<0.001**
GS	0.122	0.320	0.164	0.181	−0.046	0.707	0.527	**<0.001**	0.705	**<0.001**
GA	−0.131	0.356	−0.139	0.324	0.307	**0.027**	0.540	**<0.001**	0.613	**<0.001**
GC	/	/	0.091	0.639	/	/	/	/	0.730	**<0.001**

Note: All the correlation coefficients were calculated by partial correlation controlling for the status of sex and age. Analyses results with P < 0.05 were highlighted in bold characters.

We further investigated the correlations between PGI and PGII (Table 4). Notably, the changes of PGI expression correlated well with the changes of PGII expression no matter in situ or in serum. For the correlation between in situ PGI expression and in situ PGII expression, there were statistically significant correlations in the total study sample (Figure 5) and in the subgroups of GS and GA and a borderline correlation in NOR subgroup. For the correlation between serum PGI expression and serum PGII expression, statistically significant correlations were found in the total study sample (Figure 5) and in all the subgroups of different stomach diseases.

## Discussion

PGI and PGII are main progastricsins in the stomach, which closely reflects functional and morphological changes of gastric mucosa [15,16]. In the present study, we found that in situ levels of PGI, PGII and PGI/II ratio consistently decreased in sequence of NOR/GS- > GA- > GC, especially in GA and GC. The youngers exhibited higher levels of PGI, PGII and PGI/II ratio than the olders. Interestingly, we found statistical correlations between in situ and serum levels of PGI/II ratio in GA cases and between PGI and PGII no matter in situ or in serum. There was lack of statistical correlation between in situ and serum expressions of PGI or PGII alone in this study.

It is widely accepted that the carcinogenesis process of gastric cancer progresses stepwise from normal stomach, inflammation, precancerous conditions, and to carcinoma, as described by Correa’s cascade [17]. In the sequence of NOR/GS- > GA- > GC, consistently decreasing tendencies of in situ levels of PGI, PGII and PGI/II ratio were observed in this study. Although both NOR and GS subjects showed an extremely high positive rate of 100% of in situ PGs expression, the strongly-positive rates in GS cases significantly declined. In mild gastritis, inflammation could stimulate the production of PGs by increasing gastrin secretion; while in severe gastritis, the intensive inflammation could reversely reduce the PGs production mainly owing to injured and reduced gastric glands [18]. When it comes to GA, the positive rates of PGs expression decreased sharply, probably because the decreasing number of glands and prolonged inflammation response in GA could impair normal gland function and synthesizing capability of PGs-producing cells. Further, the synthesis function would substantially lose in intestinal metaplasia cells or cancerous cells.

A proportion of severe and extensive chronic GA could evolve into severe dysplasia and even gastric carcinoma [19]. Thus, the early diagnosis of GA is crucial for slowing down the malignant progression process of gastric mucosa. However, in clinical practice there is still certain difficulty in the early recognition of atrophy and cancerous lesions among pathologists based on haematoxylin-eosin staining of gastric biopsy. In this study, we found that only partial normal cells of remaining gastric glands in GA cases exhibited weak or moderate staining of PGI and PGII, while no PGs staining was detected in the lesions with severe atrophy, intestinal metaplasia or carcinoma. Previously, Waalewijn RA et al. [20] reported that PGI mRNA level in gastric cancer tissue was relatively low. StemmerMann GN et al. [21] showed that only 4.5% of well-differentiated intestinal-type GC and none of diffuse-type GC was PGI-positive staining. Our previous study also demonstrated that the positive rates of PGII expression decreased gradually in sequence of benign lesions, precancerous lesions and gastric cancer [12]. These observations strongly suggested that the detection of in situ PGs expression may be important auxiliary biomarkers for the recognition of the location of atrophy and carcinoma.

Serum PGs have been widely used as biomarkers for GC or GA in clinical practice [3-7]. However, the question of whether the PGs expression changes in situ are synchronistic with those in serum is still unclear. In the present study, we explored the correlations between in situ and serum expressions of PGs (including PGI, PGII and PGI/II ratio) and between PGI and PGII expressions (including in situ and in serum). However, there was lack of correlations between in situ and serum expressions of PGI or PGII in this study. One of the possible reasons for negative correlations between in situ and serum expression of PGI or PGII may be that the majority of PGs production are restricted to gastric mucosa, and only about 1% are released into blood, which may lead to nonsynchronous alterations between in situ and serum PGs expressions [1]. Another possible reason is that, apart from the influence of PGs production in stomach, the inflow of PGs from gastric epithelial cell to blood may be affected by other potential factors, such as different damage degrees of gastric epithelial cells, different vascular permeability, and different metabolic mechanisms between in situ and serum PGs [22]. Interestingly, we observed a borderline correlation in the total study sample and a statistical correlation in GA cases between in situ and serum levels of PGI/II ratio. This observation indicated that in the subjects with precancerous diseases, the serum level of PGI/II ratio, rather than serum levels of PGI or PGII alone, may be a better index that reflects the decreasing tendencies of both PGI and PGII expression in situ. This may partially explain why serum level of PGI/II ratio showed a more close correlation with GC and GA but not serum PGI or PGII alone. In addition, we found that the PGI expression correlated well with PGII expression in situ and in serum, which indicated that PGI and PGII levels change simultaneously regardless of the status of gastric mucosa. In other words, synchronous changes of PGI and PGII expressions in situ or in serum may specifically reflect the damage of gastric mucosa. However, further studies with large-scale samples are still required to validate our findings.

We are aware that there are several limitations in this study. First, the study sample size of each disease group is relatively small, which limits our stratification analysis based on different histological classifications or severity degrees. Second, only a single method was used to detect the status of *H. pylori* infection in this study population; therefore, we only evaluated the potential influence of different status of *H. pylori* serology on the in situ PGs expression. Third, in this study we only investigated PGs expression in four sequentially-evolved groups of gastric mucosa, i.e. NOR, GS, GA and GC. A longtime follow-up study of PGs expression in situ as well as in serum in the same subjects whose stomach mucosa underwent sequential changes of NOR- > GS- > GA- > GC. Further study will be required to confirm our findings and to better guide the further use of PGs in future clinical practice.

## Conclusions

In conclusion, the in situ levels of PGI, PGII and PGI/II ratio sharply decreased in the GA and GC cases. The younger people exhibited higher levels of PGI, PGII and PGI/II ratios than the older people. The in situ PGI/II ratio rather than PGI and PGII alone showed certain correlation with that in serum, and the PGI expression correlated well with PGII expression. Further studies with large-scale samples are still required to validate our findings.

## Abbreviations

GC: Gastric cancer; GS: Superficial gastritis; GA: Atrophic gastritis; NOR: Normal mucosa; PGI: Pepsinoen I; PGII: Pepsinogen II; ELISA: Enzyme-linked immunosorbent assay; IRS: Immunoreactive score; IgG: Immunoglobulin G.

## Competing interests

The authors declare that they have no competing interests.

## Authors’ contributions

PL performed the experiments and drafted the manuscript. CYH performed the statistical analysis. LPS, NND carried out the immunoassays. YY conceived of the study, and participated in its design and coordination. All authors read and approved the final manuscript.

## Pre-publication history

The pre-publication history for this paper can be accessed here:

http://www.biomedcentral.com/1472-6890/13/22/prepub
